# A phase 1 study of dalpiciclib, a cyclin-dependent kinase 4/6 inhibitor in Chinese patients with advanced breast cancer

**DOI:** 10.1186/s40364-021-00271-2

**Published:** 2021-04-12

**Authors:** Pin Zhang, Binghe Xu, Lin Gui, Wenna Wang, Meng Xiu, Xiao Zhang, Guilan Sun, Xiaoyu Zhu, Jianjun Zou

**Affiliations:** 1 National Cancer Center/Cancer Hospital, Chinese Academy of Medical Sciences and Peking Union Medical College, Beijing, China; 2 Jiangsu Hengrui Medicine Co. Ltd, Shanghai, China

**Keywords:** Cyclin-dependent kinase 4/6 inhibitor, Advanced breast cancer, First-in-human trial

## Abstract

**Background:**

Dalpiciclib (SHR6390) is a novel inhibitor of cyclin-dependent kinase 4/6 which demonstrated promising anti-tumor potency in preclinical models. This first-in-human study was conducted to evaluate the tolerability, pharmacokinetics, safety, and preliminary antitumor activity of dalpiciclib in patients with advanced breast cancer (ABC).

**Methods:**

In this open-label, phase 1 study, Chinese patients who had failed standard therapy were enrolled to receive oral dalpiciclib in 3 + 3 dose-escalation pattern at doses of 25–175 mg. Eligible patients were given a single-dose of dalpiciclib in week 1, followed by once daily continuous doses for 3 weeks, and 1 week off in 28-day cycles. Based on the tolerability, pharmacokinetics, and activity data revealed from the dose-escalation phase, three dose cohorts were selected to expand to 8–10 patients. The primary endpoints were maximum tolerated dose (MTD) and pharmacokinetics.

**Results:**

Between Apr 15, 2016 and Dec 21, 2018, 40 patients were enrolled; all were diagnosed of hormone receptor-positive and HER2-negative ABC. Dalpiciclib 100 mg, 125 mg, and 150 mg cohorts were expanded to 10 patients. No dose-limiting toxicity was observed and the MTD was not reached. Adverse events (AEs) of grade 3 or 4 were observed in 22 (55.0%) of 40 patients, being neutropenia (52.5%), leukopenia (35.0%), thrombocytopenia (5.0%), and hypertension (2.5%). No serious AEs were reported. At the doses of 50–175 mg, steady state areas under the concentration-time curve and peak concentration increased almost proportionally with dose. The disease control rate (DCR) was 62.5% (25/40, 95% CI: 45.8–77.3). Two patients (5%; 125 mg and 150 mg cohorts) achieved partial response, with responses lasting 169 and 356+ days, respectively. Among the three expansion cohorts, the 150 mg cohort had the numerically highest DCR of 80.0% (95% CI: 44.4–97.5) and longest median progression-free survival of 8.4 months (95% CI: 2.1–not reached).

**Conclusions:**

Dalpiciclib showed acceptable safety profile and dose-dependent plasma exposure in Chinese patients with ABC. The recommended phase 2 dose was 150 mg. Preliminary evidence of clinical activity was observed, which warrants further validation.

**Trial registration:**

ClinicalTrials.gov identifier: NCT02684266. Registered Feb 17, 2016.

**Supplementary Information:**

The online version contains supplementary material available at 10.1186/s40364-021-00271-2.

## Background

Cell cycle dysregulation and aberrant cell proliferation are a hallmark of cancer [1]. The cyclin D (CCND)–cyclin-dependent kinase 4/6 axis (CDK4/6), which modulates the transition through the G_1_ phase to S phase of the cell cycle, plays a key role in the pathological process of many cancer types [2]. CDK 4 and 6 can interact with CCNDs to promote the phosphorylation of the tumor-suppressor retinoblastoma protein (Rb) and the release of Rb-bound E2F transcription factor, enabling cell cycle progression from G_1_ [3]. In addition, selective CDK4/6 inhibition allows preferential inhibition of oncogenic events while sparing toxicity in normal tissues and therefore represents an appealing therapeutic strategy for cancer [3, 4].

Endocrine therapy is the cornerstone in the treatment of hormone receptor (HR)-positive advanced breast cancer (ABC). Nevertheless, acquired resistance to endocrine therapy inevitably develops during the course of treatment [5]. Estrogen-mediated hyperactivity of the CCND–CDK4/6 axis is a central feature of HR-positive breast cancer, and the tumors usually retain a functional Rb [6], which can be targeted by CDK4/6 inhibitors. To date, three CDK4/6 inhibitors including palbociclib, ribociclib, and abemaciclib have been approved by the US Food and Drug Administration and the European Medicines Agency in combination with endocrine therapy as the first- and second-line treatment for HR-positive and human epidermal growth factor receptor 2 (HER2)-negative ABC. In pivotal trials, the addition of these CDK4/6 inhibitors to standard endocrine therapy substantially improved progression-free survival (PFS) and overall survival [7–15]. Abemaciclib has also been approved as a monotherapy for HR-positive and HER2-negative breast cancer progressing on prior endocrine therapy or chemotherapy in the metastatic setting [16].

Dalpiciclib (SHR6390) is a novel, highly selective, small molecule CDK4/6 inhibitor with comparable potencies against CDK4 (IC_50_, 12.4 nM) and CDK6 (IC_50_, 9.9 nM). Dalpiciclib has demonstrated anti-tumor activity in a variety of in vitro and xenograft models primarily via Rb-dependent cytostasis [17, 18]. In vivo xenografts, dalpiciclib generally showed similar or slightly better anti-tumor potency compared with palbociclib, without inducing noticeable toxicity [18]. Moreover, in HR-positive breast cancer cell lines and xenografts, dalpiciclib could overcome the acquired drug resistance to endocrine therapy [18]. Based on these preclinical evidence, we conducted a first-in-human, phase 1 trial to assess the safety, tolerability, pharmacokinetics (PK) and preliminary efficacy of oral dalpiciclib in patients with HR-positive and HER2-negative ABC.

## Methods

### Study design

**Fig. 1:**
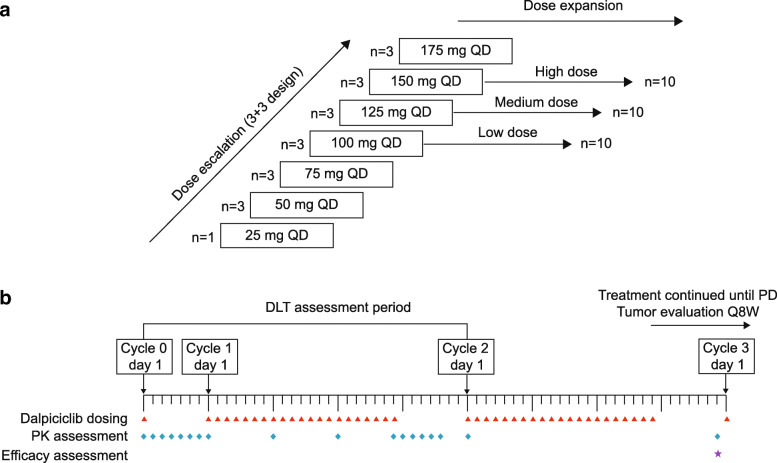
Study design. **a** Framework for the dose-escalation and dose-expansion study of dalpiciclib. **b** Dosing and assessment schema. DLT, dose-limiting toxicity; PD, progressive disease

This open-label, phase 1 trial in patients with ABC was conducted in China (ClinicalTrials.gov identifier: NCT02684266). The study consisted of dose-escalation and dose-expansion phases. In the dose-escalation phase, the primary objective was to establish the maximum tolerated dose (MTD) for oral dalpiciclib. In the dose-expansion phase, three selected dose cohorts were expanded to further characterize the safety profile, tolerability, PK parameters and preliminary anti-tumor activity of dalpiciclib (Fig. 1).

### Patients

Eligible patients were aged 18–65 years, with histologically confirmed ABC who failed standard therapy. Other inclusion criteria included Eastern Cooperative Oncology Group performance status 0–1, life expectancy ≥3 months, adequate bone marrow function (hemoglobin > 110 g/L, neutrophils > 2.0 × 10^9^ per L and platelets > 100 × 10^9^ per L), adequate liver and renal function (total bilirubin ≤ 1.5 times the upper limit of normal [ULN], alanine aminotransferase [ALT] and aspartate aminotransferase [AST] ≤ 1.5 times ULN [≤ 5 times ULN in the presence of liver metastases] and creatinine ≤1 ULN), and adequate cardiac function (left ventricular ejection fraction ≥50% and Fridericia corrected-QT [QTcF] interval < 450 ms in males or < 470 ms in females). The key exclusion criteria were prior or current treatment with CDK4/6 targeted therapy, cytotoxic chemotherapy within 3 weeks (6 weeks for mitomycin C or nitrosamine) or any other anti-tumor therapy within 3 weeks (except for endocrine therapy, which had to be discontinued before the time of informed consent), untreated or uncontrolled/unstable brain metastases (as judged by the investigator) or requirement of long-term use of steroids.

### Procedures

For dose escalation, a traditional 3 + 3 design was used with three to six patients enrolled per dose level and the escalation continued until two or more patients had dose-limiting toxicities (DLTs) in one dose cohort during the DLT assessment period (from the administration of the first study dose to the end of the first cycle). The starting dose of dalpiciclib was 25 mg and was escalated by 25 mg increments up to 175 mg (25 mg, 50 mg, 75 mg, 100 mg, 125 mg, 150 mg and 175 mg) in a modified Fibonacci schema (Fig. 1a). At the 25 mg dose level, one patient was enrolled as a sentinel; if no grade ≥ 2 toxicity was observed during the sentinel patient’s DLT assessment period, escalation could proceed to the 50 mg dose level immediately. DLT was defined as an adverse event (AE) that met any of the following criteria: grade 4 hematologic toxicity, grade 3 neutropenia with infection or fever ≥38.5 °C, grade 3 thrombocytopenia with apparent clinical bleeding tendency, grade ≥ 3 non-hematologic toxicity (excluding untreated nausea, vomiting, and diarrhea or AEs considered tolerable by the patients, such as alopecia) or grade ≥ 2 cardiac or renal toxicity. Based on the tolerability, PK, and anti-tumor activity revealed from the dose-escalation phase, three dose cohorts were selected to expand to 8–10 patients.

All patients were first given a single dose of oral dalpiciclib. After a ≥ 7-day washout period, the patient was then administered once-daily continuous doses of dalpiciclib for 3 weeks in 28-day cycles (Fig. 1b). Dose interruption or reduction was permitted after the initiation of the second cycle of treatment as prespecified in protocol (Additional file 1: Table S1). Patients who achieved complete response [CR], partial response [PR] or stable disease [SD]) at first efficacy assessment after receiving two cycles of dalpiciclib could continue treatment until disease progression, intolerable toxicity, withdrawal of patient consent or investigator decision.

### Endpoint

The primary endpoint was the MTD of dalpiciclib and pharmacokinetic parameters in patients with ABC. The MTD was the highest dose level at which < 33% of the patients reported a DLT within the first cycle of multiple dosing. The secondary endpoints included safety and the investigator-assessed objective response rate (ORR, defined as the proportion of patients with CR and PR as the best overall response) and disease control rate (DCR, defined as the proportion of patients with CR, PR or SD ≥ 6 weeks) per RECIST v1.1.

### Assessment

Safety was regularly monitored from the time of informed consent until 30 days after the last dose of study drug. AEs were graded according to the Common Terminology Criteria for Adverse Events v4.0. Tumor response was assessed using computerized tomography or magnetic resonance imaging at baseline and every two cycles after the start of multiple once-daily dosing. The response was classified as CR, PR, SD or progressive disease (PD) according to RECIST v1.1 by the investigators [19]. A CR or PR was required to be confirmed with a subsequent scan 4 weeks after the initial assessment.

For PK analysis, blood samples were collected in all enrolled patients from predose and at intervals up to 24 h (0.5 h, 1 h, 2 h, 3 h, 4 h, 6 h, 8 h, 10 h and 24 h) on day 1 and every 24 h thereafter through day 6 at the single-dose stage; and on day 1 (predose), day 8 (predose), day 15 (predose), day 21 (predose and 0.5 h, 1 h, 2 h, 3 h, 4 h, 6 h, 8 h and 10 h postdose) and day 22 through day 26 (every 24 h) of cycle 1 at the multiple-dose stage. The plasma concentrations of dalpiciclib were measured by liquid chromatography with tandem mass spectrometry.

### Statistics

The DLT analysis set included all patients who received at least one dose of the study drug and completed the first cycle of treatment or discontinued study drug due to AE during the first cycle of treatment. PK was analyzed in patients who received at least one dose of the study drug and had evaluable post-treatment PK data. Safety and efficacy were analyzed in all patients who received at least one dose of the study drug. Descriptive statistics were reported for the safety and efficacy outcomes and PK parameters. The ORR and DCR were calculated, with the corresponding 95% Clopper-Pearson confidence intervals (CIs) provided. The Kaplan-Meier method was used to analyze PFS, and the median PFS (month) was estimated. The two-sided 95% CI of the median PFS was calculated by the Brookmeyer Crowley method. PK parameters were analyzed using the non-compartmental model with WinNonlin 7.0 (Pharsight, Mountain View, CA). Statistical analyses were conducted with SAS 9.4 (SAS Institute Inc., Cary, NC).

## Results

### Patient characteristics and deposition

Between Apr 15, 2016 and Dec 21, 2018, 58 patients were screened. Among them, 40 patients were enrolled and received dalpiciclib. All patients were diagnosed of HR-positive and HER2-negative stage IV ABC. 45.0% of the patients had at least three lines of prior chemotherapies and 55.0% had at least two prior endocrine therapies (Table 1). The median follow-up was 7.0 months (range, 2.7–35.0). The main reason for treatment discontinuation was disease progression (28 patients, 70.0%). At data cutoff (Jun 28, 2019), treatment was still on-going in 10 patients (25.0%). No deaths were reported.

**Table 1:** Demographics and baseline disease characteristics

	25 mg (*n* = 1)	50 mg (*n* = 3)	75 mg (*n* = 3)	100 mg (*n* = 10)	125 mg (*n* = 10)	150 mg (*n* = 10)	175 mg (*n* = 3)	All patients (*n* = 40)
Age, years	50 (50–50)	52 (51–64)	53 (48–63)	58 (39–65)	54 (26–63)	55 (39–65)	51 (51–64)	54 (26–65)
Sex								
Female	1 (100)	3 (100)	3 (100)	10 (100)	10 (100)	10 (100)	3 (100)	40 (100)
ECOG performance status								
0	1 (100)	3 (100)	2 (66.7)	4 (40.0)	10 (100)	9 (90.0)	3 (100)	32 (80.0)
1	0	0	1 (33.3)	6 (60.0)	0	1 (10.0)	0	8 (20.0)
Time since first diagnosis, years	10.9 (10.9–10.9)	5.2 (1.7–11.9)	5.9 (2.4–12.0)	9.6 (4.9–20.3)	6.8 (1.4–20.1)	11.3 (1.0–19.6)	8.2 (8.2–10.5)	8.8 (1.0–20.3)
Tumor stage at study entry								
IV	1 (100)	3 (100)	3 (100)	10 (100)	10 (100)	10 (100)	3 (100)	40 (100)
Previous therapy								
Surgery	1 (100)	3 (100)	3 (100)	10 (100)	10 (100)	10 (100)	3 (100)	40 (100)
Radiotherapy	1 (100)	3 (100)	3 (100)	8 (80.0)	5 (50.0)	8 (80.0)	3 (100)	31 (77.5)
Chemotherapy								
1–2 regimens	0	0	2 (66.7)	2 (20.0)	4 (40.0)	3 (30.0)	2 (66.7)	13 (32.5)
3–6 regimens	1 (100)	0	1 (33.3)	6 (60.0)	4 (40.0)	5 (50.0)	1 (33.3)	18 (45.0)
Endocrine therapy								
1–2 regimens	0	1 (33.3)	2 (66.7)	4 (40.0)	4 (40.0)	6 (60.0)	3 (100)	20 (50.0)
3–6 regimens	1 (100)	0	1 (33.3)	3 (30.0)	3 (30.0)	3 (30.0)	0	11 (27.5)

Data are n (%) or median (range)

*ECOG* Eastern Cooperative Oncology Group

### Safety and tolerability

Dalpiciclib was dose escalated from 25 mg QD to 175 mg QD. No DLT was observed in all dose cohorts within the first cycle of treatment and the MTD was not reached. With PK analysis showing increased exposure (C_max_, C_min_, AUC_ss_) of dalpiciclib and increased occurrence of neutropenia and leukopenia (indicator of pharmacodynamics) within first cycle at doses of 50–150 mg, and the best overall response of SD or better for all patients at 100–150 mg (4/7 PD at 25–75 mg and 1/3 PD at 175 mg), 150 mg was first selected for expansion. Then with safety as a main consideration (8/10 with grade 3 and 1/10 with grade 4 neutropenia for the 150 mg cohort), the 125 mg and 100 mg cohorts were subsequently expanded to 10 patients. Overall, no DLT was observed in the expansion cohorts within the prespecified assessment window.

The median duration of treatment exposure was 123 days (range, 56–1063). No patients discontinued treatment due to AEs. All patients in the study had at least one treatment-emergent AE (TEAE). Common TEAEs and all TEAEs occurring in ≥2 patients are provided in Table 2 and Additional file 1 Table S2 respectively. The most frequent non-hematological toxicities were increased AST (40.0%) and ALT (32.5%); of these, 2 cases of increased AST and 1 case of increased ALT were of grade 2 severity and all others were of grade 1. QT prolongation was reported for 5 (12.5%) patients, including 3 with a maximum QTcF interval of < 480 ms (grade 1) and 2 with a maximum interval of 480–500 ms (grade 2). No patient had a maximum value of > 500 ms or a > 60 ms increase from baseline. Among the 5 cases with QT prolongation, 2 (5%; both grade 1) were considered possibly treatment-related by the investigators. Thrombosis (grade 1) was reported in 1 patient and judged as treatment unrelated. TEAEs of grade 3 or 4 were observed in 22 (55.0%) of 40 patients, being neutropenia (52.5%), leukopenia (35.0%), thrombocytopenia (5.0%), and hypertension (2.5%). No serious AEs or grade 5 AEs were reported.

**Table 2:** Treatment-emergent adverse events (TEAEs) occurring in ≥20% of patients

	All patients (*n* = 40)		
	All Grades	Grade 3	Grade 4
Hematologic TEAEs			
Neutropenia	40 (100)	19 (47.5)	2 (5.0)
Leukopenia	40 (100)	13 (32.5)	1 (2.5)
Anemia	13 (32.5)	0	0
Thrombocytopenia	11 (27.5)	1 (2.5)	1 (2.5)
Non-hematologic TEAEs			
Aspartate aminotransferase increased	16 (40.0)	0	0
Fatigue	15 (37.5)	0	0
Blood creatinine increased	14 (35.0)	0	0
Alanine aminotransferase increased	13 (32.5)	0	0
Headache	13 (32.5)	0	0
Alopecia	11 (27.5)	0	0
Bilirubin conjugated increased	10 (25.0)	0	0
Decreased appetite	9 (22.5)	0	0
Constipation	8 (20.0)	0	0
Dyspnoea	8 (20.0)	0	0

Data are n (%). No grade 5 adverse events occurred

### PK

PK parameters following single and multiple dosing of dalpiciclib are presented in Table 3 and the plasma concentration-time curves are shown in Additional file 1 Figure S1. At doses of 50–175 mg, the median time to peak concentration was 2.5–4.0 h, and the geometric mean terminal half-life was 40.3–51.4 h after a single dose of dalpiciclib. Following multiple dosing, steady state dalpiciclib was observed on day 8. The median time to peak concentration was 3.0–4.0 h, and the geometric mean terminal half-life was 44.9–52.3 h at steady state on day 21 and the mean accumulation ratio for area under the concentration-time curve was 1.9–3.4 across the doses of 50–175 mg. The area under the concentration-time curve at steady state and peak concentration increased almost proportionally with dose increase over the dosing range of 50–175 mg. The steady state C_max_ at day 21 was 41.1, 53.4, 87.0, 115, 126, and 155 ng/mL in the 50, 75, 100, 125, 150, and 175 mg cohorts, respectively (Table 3).

**Table 3:** Plasma pharmacokinetics of single and multiple dosing of dalpiciclib

	50 mg QD	75 mg QD	100 mg QD	125 mg QD	150 mg QD	175 mg QD
Single dosing	*n* = 3	*n* = 3	*n* = 10	*n* = 10	*n* = 10	*n* = 3
T_max_, h	3.0 (2.0–4.0)	3.0 (3.0–3.0)	2.5 (2.0–4.0)	3.5 (2.0–6.0)	3.5 (2.0–4.0)	4.0 (3.0–4.0)
C_max_, ng/mL	12.2 (57.3)	20.3 (37.3)	46.8 (27.5)	46.6 (56.8)	63.6 (64.4)	114 (37.8)
AUC_0-t_, h*ng/mL	482 (40)	572 (13)	1170 (20)	1480 (38)	1880 (57)	3080 (24)
t_1/2z_, h	51.0 (13.6)	46.9 (8.0)	49.7 (16.6)	43.7 (23.5)	51.4 (23.2)	40.3 (22.4)
CL/F, L/h	89.8 (39.2)	117 (15.7)	76.1 (20.7)	76.6 (37.8)	70.5 (51.9)	52.8 (21.8)
V_z_/F, L	6610 (40)	7900 (12)	5450 (25)	4830 (45)	5230 (71)	3080 (39)
Multiple dosing	*n* = 3	*n* = 3	*n* = 9	*n* = 9	*n* = 9	*n* = 3
T_max_, h	4.0 (4.0–6.0)	3.0 (3.0–4.0)	3.0 (2.0–6.0)	4.0 (2.0–6.0)	4.0 (2.0–8.0)	3.0 (3.0–3.0)
C_max_, ng/mL	41.1 (23.9)	53.4 (9.4)	87.0 (48.5)	115 (28.7)	126 (30.0)	155 (57.8)
AUC_ss_, h*ng/mL	615 (27)	792 (18)	1410 (53)	2020 (32)	2230 (24)	2730 (48)
t_1/2z_, h	46.9 (11.9)	52.3 (6.3)	48.2 (20.3)	44.9 (21.2)	44.9 (17.5)	45.1 (6.6)
CL_ss_/F, L/h	81.4 (26.9)	94.7 (18.0)	71.0 (52.5)	62.0 (31.8)	67.2 (23.9)	64.1 (47.8)
V_z_/F, L	5510 (35)	6920 (31)	4930 (67)	4010 (30)	4350 (32)	4170 (48)
R_acc(AUC)_	3.4 (41.3)	3.0 (22.1)	2.7 (35.9)	3.3 (60.0)	2.7 (52.8)	1.9 (37.7)

Data are median (range) for T_max_ and geometric mean (geometric coefficient of variation%) for others

*T*_*max*_ time to reach C_max_, *C*_*max*_ peak plasma concentration, *AUC*_*0-t*_ area under the curve from time 0 to the last measurable concentration, *AUC*_*ss*_ area under the curve for dose interval, *t*_*1/2*_ terminal half-life, *CL/F* apparent clearance, *V*_*z*_*/F* apparent volume of distribution, *R*_*ac (AUC)*_ accumulation ratio for AUC

### Efficacy

All 40 patients with HR-positive and HER2-negative ABC were evaluable for tumor response. Of those, two patients (5%) achieved PR: one patient (125 mg cohort) had previously received two lines of chemotherapy for ABC and partial response was initially observed at cycle-8 tumor assessment visit and lasted for 169 days; the other patient (150 mg cohort) had previously received six regimens of endocrine therapy and partial response was initially documented at cycle-4 tumor assessment visit and lasted for 356+ days. A total of 23 (57.5%) patients across 50–175 mg dose cohorts had best overall response with stable disease and the median duration was 4.0 months (inter-quartile range, 2.1–7.6). The overall DCR was 62.5% (95% CI: 45.8–77.3) in all patients (Table 4). By data cutoff, a total of 26 disease progression events occurred. Median PFS was 4.0 months (95% CI: 2.4–9.1) in all 40 patients and 5.9 months (95% CI: 2.4–9.1) in 30 patients at the three dose levels with expansion cohorts. Among the three dose cohorts with expansion, the 150 mg cohort had the numerically highest DCR of 80.0% (95% CI: 44.4–97.5) and longest median PFS of 8.4 months (95% CI: 2.1–not reached).

**Table 4:** Tumor response per RECIST v1.1

	25 mg (*n* = 1)	50 mg (*n* = 3)	75 mg (*n* = 3)	100 mg (*n* = 10)	125 mg (*n* = 10)	150 mg (*n* = 10)	175 mg (*n* = 3)	All patients (*n* = 40)
Best overall response, n (%)								
Complete response	0	0	0	0	0	0	0	0
Partial response	0	0	0	0	1 (10.0)	1 (10.0)	0	2 (5.0)
Stable disease	0	2 (66.7)	1 (33.3)	7 (70.0)	4 (40.0)	7 (70.0)	2 (66.7)	23 (57.5)
Progressive disease	1	1 (33.3)	2 (66.7)	3 (30.0)	5 (50.0)	2 (20.0)	1 (33.3)	15 (37.5)
DCR, % (95% CI)	0 (NR)	66.7 (9.4–99.2)	33.3 (0.8–90.6)	70.0 (34.8–93.3)	50.0 (18.7–81.3)	80.0 (44.4–97.5)	66.7 (9.4–99.2)	62.5 (45.8–77.3)

*DCR* disease control rate, *NR* not reached, *RECIST* response evaluation criteria in solid tumors

## Discussion

The findings from this first-in-human, phase 1 trial showed that oral dalpiciclib with a 3-week on and 1-week off dosing regimen was well-tolerated in patients with ABC, with no MTD observed up to the highest dose tested. TEAEs were generally manageable with no treatment discontinuation due to AEs or serious AEs documented. At doses of 50–175 mg, plasma exposure increased almost proportionally with dose. Preliminary evidence of clinical activity of dalpiciclib in HR-positive and HER2-negative ABC was also observed, with a DCR of 62.5%.

Dalpiciclib was rapidly absorbed and reached C_max_ within 2.5–4.0 h upon intake. No saturation of absorption was observed at doses of 50–175 mg; together with the terminal half-life of 40–51 h, the PK data of dalpiciclib supported the once-daily administration schedule. Dalpiciclib showed a large apparent volume of distribution (3080–7900 L), suggesting that it was widely distributed in the body. In our preclinical tissue distribution study in rats, [^14^C] dalpiciclib was found widely distributed in all tissues with limited brain penetration (unpublished data). In vivo study has showed that dalpiciclib was a P-gp substrate. Since membrane transporters at the blood brain barrier are involved in the regulation of brain penetration of CDK4/6 inhibitors, their interaction with dalpiciclib requires further investigation [20]. Similar to other CDK4/6 inhibitors, dalpiciclib was mainly metabolized in the liver via CYP3A4. In addition, CYP2C9 and CYP2C8 also mediated part of the metabolism of dalpiciclib. Therefore, the exposure of dalpiciclib might be affected by inhibitors or inducers of these enzymes. In vivo drug-drug interaction studies with itraconazole, rifampin and other drugs are underway and the results will provide guidance on clinical concomitant medications. The pharmacokinetic characteristics of dalpiciclib and other approved CDK4/6 inhibitors are provided in detail in Additional file 1 Table S3.

The definitions of DLT and MTD used in our study were accordant with those previously used in studies on palbociclib [21]. During dose-escalation and expansion, no DLT was observed in all patients within the first cycle of treatment. In the phase 1 dose-escalation study of palbociclib, DLT was reported in 5 (12%) patients, all being neutropenia and the MTD and the recommended phase 2 dose was established as 125 mg [21]. In the phase 1 study of ribociclib, DLTs were reported in 9 (13%) patients within the first treatment cycle, including 3 cases of neutropenia, 2 cases of thrombocytopenia, and 1 each of mucositis, pulmonary embolism, QTcF prolongation and increased creatinine [22]. Consistent with the safety profile of other CDK4/6 inhibitors with similar potencies for CDK4 and CDK6 (palbociclib and ribociclib) [22–24], the most frequent grade 3 or 4 AEs of dalpiciclib were neutropenia and leukopenia, observed in 52.5 and 35.0% of patients respectively. All the hematologic AEs were managed with dose interruption and/or reduction and standard supportive care (granulocyte colony-stimulating factor etc.). The abnormalities resolved immediately with no related complications such as fever or infection observed. The myelosuppressive effects of dalpiciclib were considered an on-target effect due to the involvement of CDK6 in the proliferation of hematological precursors [25] and the cytostasis induced in G_1_ phase by dalpiciclib was reversible when the drug was held. Abemaciclib shows a different safety profile with higher gastrointestinal toxicity and less hematological toxicity, which might be attributed to its higher potency against CDK4 than CDK6 and additional potency against CDK9 [9, 26, 27]. Of note, the incidence of increased AST and ALT were observed in 40 and 32.5% of patients respectively in this study, probably due to the high prevalence of liver metastases at baseline (47.5%; 19/40) and patient fragility from heavy pretreatment (45.0% patients with ≥3 prior lines of chemotherapy, 27.5% with ≥3 prior lines of endocrine therapy). However, the hepatotoxicity was mild with only 3 cases of grade 2 and no case of grade ≥ 3 documented. QTcF prolongation was previously reported in 6–9% patients with ribociclib [8, 22]; in the phase 1 trial of palbociclib, 26 of 41 patients had a maximum increase from baseline QTc of < 30 ms, but none had a QTc value of > 500 ms [21]. Prolongation of the QT interval was observed in 12.5% (all with a maximum value of < 500 ms) of patients during treatment with dalpiciclib; nevertheless, only 2 cases were considered treatment-related and both resolved without medical intervention. Lung inflammation, a rare but serious adverse effect reported for other CDK4/6 inhibitors [28], was not observed in the present study, possibly due to the relative small sample size. Taken together, dalpiciclib with a 3-week on/1-week off dosing regimen is well-tolerated with a manageable safety profile in patients with ABC.

Despite the initial success of endocrine therapy in the treatment of HR-positive and HER2-negative ABC, nearly all patients will develop acquired drug resistance with prolonged treatment [5]. In this phase 1 trial, clinical activity of single agent dalpiciclib in HR-positive and HER2-negative ABC was demonstrated with an overall DCR of 62.5% (95% CI: 45.8–77.3) across all dose levels, consistent with the preclinical evidence that dalpiciclib could overcome the resistance to an endocrine agent (tamoxifen) [18]. Despite the modest ORR rate (5%), dalpiciclib was notable for the durable disease control, with a median duration of 4.0 months. Among all therapeutic dose levels with an expansion cohort (100–150 mg), patients treated with 150 mg QD dalpiciclib appeared to derive the greatest clinical benefit, with a DCR of 80.0% (95% CI: 44.4–97.5) and a median PFS of 8.4 months. Taken together with the dose-dependent increase in systemic exposure of dalpiciclib (C_max_, C_min_, AUC_ss_) and the overall tolerability at doses of 100–150 mg, 150 mg QD was selected as the recommended phase 2 dose for ABC. In previous phase 2 trials of CDK4/6 inhibitors on HR-positive and HER2-negative metastatic breast cancer, treatment with single agent abemaciclib yielded a DCR of 66.7% and a median PFS of 5.9 months in a heavily pretreated population (median prior lines of therapy, 2 for endocrine agents, 1 for chemotherapy) whereas single agent palbociclib yielded a similar DCR of 63.8% and a median PFS of 6.5 months in a moderately pretreated population (69% with 1 line of prior endocrine therapy, 28% with prior chemotherapy) [16, 24]. Within the limitations of cross-trial comparison, the anti-tumor activity of single agent dalpiciclib reported in this trial was comparable to abemaciclib and palbociclib in treatment of ABC. In general, our results were encouraging considering that the efficacy data of dalpiciclib were derived from a heavily pretreated population with refractory disease and from single agent treatment. Given the established synergic effects of dalpiciclib with letrozole, anastrozole and fulvestrant in a phase Ib trial (NCT03481998, Hengrui data on file) and the purpose to further improve anti-tumor activities, dalpiciclib is currently under evaluation in combination with endocrine therapy for HR-positive and HER2-negative ABC in two phase 3 trials: one in combination with letrozole or anastrozole in the frontline setting (NCT03966898) and one in combination with fulvestrant in the later-line setting (NCT03927456).

In summary, dalpiciclib showed acceptable safety profile and dose-dependent plasma exposure in Chinese patients with ABC. The recommended phase 2 dose was determined to be 150 mg QD. Preliminary evidence of clinical activity of dalpiciclib was observed.

## Supplementary Information

**Additional file 1: **Online supplemental figures and tables.docx. **Figure S1.** Linear plot of concentration-time profile for dalpiciclib by dose. **Table S1.** Dose modifications after DLT assessment period. **Table S2.** Treatment emergent AEs occurring in ≥2 patients and AEs of special interest. **Table S3.** Pharmacokinetics and pharmacological characteristics of CDK4/6 inhibitors.

## Data Availability

All data generated or analyzed during this study are included in this published article and its supplementary information files.

## Abbreviations

ABC: Advanced breast cancer
AE: Adverse event
ALT: Alanine aminotransferase
AST: Aspartate aminotransferase
CI: Confidence interval
CCND: Cyclin D
CDK4/6: Cyclin-dependent kinase 4/6
CR: Complete response
DCR: Disease control rate
DLT: Dose-limiting toxicity
HER2: Human epidermal growth factor receptor 2
HR: Hormone receptor
MTD: Maximum tolerated dose
ORR: Objective response rate
PD: Progressive disease
PFS: Progression-free survival
PK: Pharmacokinetics
PR: Partial response
Rb: Retinoblastoma protein
SD: Stable disease
TEAE: Treatment-emergent adverse event
ULN: Upper limit of normal
