# Supplementary material for: A phase 1 study of dalpiciclib, a cyclin-dependent kinase 4/6 inhibitor in Chinese patients with advanced breast cancer
Source: Biomark Res. 2021 Apr 12;9:24. doi: 10.1186/s40364-021-00271-2 (PMC8042970; doi:10.1186/s40364-021-00271-2)
Supplement: Supplementary file 1 — Additional file 1: Online supplemental figures and tables.docx. Figure S1. Linear plot of concentration-time profile for dalpiciclib by dose. Table S1. Dose modifications after DLT assessment period. Table S2. Treatment emergent AEs occurring in ≥2 patients and AEs of special interest. Table S3. Pharmacokinetics and pharmacological characteristics of CDK4/6 inhibitors. [file 40364_2021_271_MOESM1_ESM.docx]

**Additional file 1**

**

**

**Figure S1**. Linear plot of concentration-time profile for dalpiciclib by dose. (A) Plasma

pharmacokinetics after a single dose of oral dalpiciclib (n=40); (B) Plasma pharmacokinetics after once daily oral administration of dalpiciclib on cycle 1 day 21 (n=36). Data are mean ± standard deviation.

**Table S1.** Dose modifications after DLT assessment period^*^

|  | Severity (Per CTCAE version 4.0) | | | |
| --- | --- | --- | --- | --- |
| AE | Grade 1 | Grade 2 | Grade 3 | Grade 4 |
| Hematological toxicity | Maintain dose | Maintain dose | Interrupt dose until symptoms resolve to grade ≤1^†^;  then maintain dose or reduce dose by 1 level per investigator's decision^‡^ | Interrupt dose until symptoms resolve to grade ≤1^†^;  then reduce dose by 1 level per investigator's decision^‡^ |
| Non-hematological toxicity | Maintain dose | Interrupt dose until symptoms resolve to grade ≤1^†^;  then maintain dose or reduce dose by 1 level per investigator's decision^‡^ | Interrupt dose until symptoms resolve to grade ≤1^†^;  then reduce dose by 1 level per investigator's decision^‡^ | Permanently discontinue |
| Febrile Neutropenia | - | - | Interrupt dose until symptoms resolve to grade ≤1^†^;  then reduce dose by 1 level per investigator's decision^‡^ | Permanently discontinue |

^*^Per protocol, during DLT assessment period (ie. cycle 0 and 1), non-DLTs were not medically managed to assess the reversibility of AEs; in case of DLTs, patients were actively managed and treatment was discontinued.

^†^Dose interruption up to 2 weeks were allowed.

^‡^1 dose level=25 mg

DLT, dose limiting toxicity.

**Table S2.** Treatment emergent AEs occurring in ≥ 2 patients and AEs of special interest

|  | All patients (n=40) | | |
| --- | --- | --- | --- |
|  | All Grades | Grade 3 | Grade 4 |
| Neutropenia | 40 (100) | 19 (47.5) | 2 (5.0) |
| Leukopenia | 40 (100) | 13 (32.5) | 1 (2.5) |
| Aspartate aminotransferase increased | 16 (40.0) | 0 | 0 |
| Fatigue | 15 (37.5) | 0 | 0 |
| Blood creatinine increased | 14 (35.0) | 0 | 0 |
| Alanine aminotransferase increased | 13 (32.5) | 0 | 0 |
| Headache | 13 (32.5) | 0 | 0 |
| Anaemia | 13 (32.5) | 0 | 0 |
| Thrombocytopenia | 11 (27.5) | 1 (2.5) | 1 (2.5) |
| Alopecia | 11 (27.5) | 0 | 0 |
| Bilirubin conjugated increased | 10 (25.0) | 0 | 0 |
| Decreased appetite | 9 (22.5) | 0 | 0 |
| Constipation | 8 (20.0) | 0 | 0 |
| Dyspnoea | 8 (20.0) | 0 | 0 |
| Diarrhoea | 7 (17.5) | 0 | 0 |
| Nausea | 6 (15.0) | 0 | 0 |
| Stomatitis | 6 (15.0) | 0 | 0 |
| Electrocardiogram QT prolonged | 5 (12.5) | 0 | 0 |
| Blood bilirubin increased | 5 (12.5) | 0 | 0 |
| Blood bilirubin unconjugated increased | 5 (12.5) | 0 | 0 |
| Mouth ulceration | 5 (12.5) | 0 | 0 |
| Toothache | 4 (10.0) | 0 | 0 |
| Pruritus | 4 (10.0) | 0 | 0 |
| Cough | 4 (10.0) | 0 | 0 |
| White blood cell count increased | 3 (7.5) | 0 | 0 |
| Asthenia | 3 (7.5) | 0 | 0 |
| Influenza like illness | 3 (7.5) | 0 | 0 |
| Bacteriuria | 3 (7.5) | 0 | 0 |
| Myalgia | 3 (7.5) | 0 | 0 |
| Palpitations | 3 (7.5) | 0 | 0 |
| Sinus tachycardia | 3 (7.5) | 0 | 0 |
| Hypertension | 3 (7.5) | 1 (2.5) | 0 |
| Gamma-glutamyltransferase increased | 2 (5.0) | 0 | 0 |
| Weight decreased | 2 (5.0) | 0 | 0 |
| Blood potassium decreased | 2 (5.0) | 0 | 0 |
| Blood alkaline phosphatase increased | 2 (5.0) | 0 | 0 |
| Blood glucose increased | 2 (5.0) | 0 | 0 |
| Neutrophil count increased | 2 (5.0) | 0 | 0 |
| Vomiting | 2 (5.0) | 0 | 0 |
| Abdominal pain upper | 2 (5.0) | 0 | 0 |
| Pyrexia | 2 (5.0) | 0 | 0 |
| Rash maculo-papular | 2 (5.0) | 0 | 0 |
| Hypoaesthesia | 2 (5.0) | 0 | 0 |
| Dizziness | 2 (5.0) | 0 | 0 |
| Nasopharyngitis | 2 (5.0) | 0 | 0 |
| Pain in extremity | 2 (5.0) | 0 | 0 |
| Other AEs of special interest |  |  |  |
| Thrombosis | 1 (2.5) | 0 | 0 |

Data are n (%). No grade 5 adverse events occurred. AE, adverse events.

**Table S3.** Pharmacokinetics and pharmacological characteristics of CDK4/6 inhibitors^*^

|  | Dalpiciclib | Palbociclib | Ribociclib | Abemaciclib |
| --- | --- | --- | --- | --- |
| Route of administration | Oral | Oral | Oral | Oral |
| Recommended dose (mg) | 150 q.d. | 125 q.d. | 600 q.d. | 200 b.i.d. |
| Schedule | 3 wks on/1 wk off | 3 wks on/1 wk off | 3 wks on/1 wk off | Continuous |
| Metabolism | Liver  (CYP3A/CYP2C9/CYP2C8) | Liver  (CYP3A/SULT2A1) | Liver  (CYP3A4) | Liver  (CYP3A4) |
| CDK4 IC_50_ (nM) | 12.4 | 9–11 | 8 | 2 |
| CDK6 IC_50_ (nM) | 9.9 | 15 | 39 | 9.9 |
| T_max_ (h) | 4.0 (2.0−8.0) | 6−12 | 1−4 | 8 (4.1−24) |
| t_1/2_ (h) | 44.9 | 29 | 32 | 18.3 |
| Volume of distribution (L) | 4350 | 2583 | 1090 | 690.3 |
| C_min,ss_ (ng/mL) | 66.8 | 61 | 631 | 197 |
| AUC_ss_ (h*ng/mL) | 2230 | 1633-1982 | 22700 | 3000 |
| Accumulation ratio | 2.71 | 2.4 | 2.51 | 2.53 |
| Excretion (%) |  |  |  |  |
| Feces | 72 | 74 | 69 | 81 |
| Urine | 23 | 18 | 23 | 3 |
| Mild hepatic/renal impairment | NA | No effect on exposure | No effect on exposure | No effect on exposure |

^*^Data on palbociclib, ribociclib and abemaciclib are based on product label or FDA review.

AUC_ss_: area under the curve for dose interval; C_min,ss_, minimum steady-state plasma drug concentration during a dosage interval; NA, not available; T_max_, time to reach peak plasma concentration; t_1/2_, terminal half-life.
